# India Leads the Way: A Health-Centered Strategy for Air Pollution

**DOI:** 10.1289/EHP90

**Published:** 2016-07-01

**Authors:** Ambuj Sagar, Kalpana Balakrishnan, Sarath Guttikunda, Anumita Roychowdhury, Kirk R. Smith

**Affiliations:** 1 Indian Institute of Technology Delhi, New Delhi, India; 2 Sri Ramachandra University, Chennai, Tamil Nadu, India; 3 Centre for Science and Environment, New Delhi, India; 4 University of California, Berkeley, Berkeley, California, United States

The Government of India has recently initiated unprecedented efforts to address the substantial national health burden attributable to ambient and household air pollution. The key first step was the constitution by the Ministry of Health and Family Welfare (MHFW) of an expert committee on air pollution and health. This committee put together a landmark report (MHFW 2015) released earlier this year that outlined targeted actions aimed at providing the largest exposure reductions (and consequent health benefits), instead of traditional approaches to air quality management. India’s health ministry is perhaps the first among low- and middle-income countries to initiate steps that directly address air pollution as a national health concern. Discussions initiated through this process have led to a number of concrete policy actions consistent with these recommendations, the most recent of which is highlighted within the 2016–2017 budget just released by the government: a commitment of at least US$1.5 billion to address household air pollution by providing clean cooking gas to 50 million poor households in the next 3 years (MF 2016). This is an enormous step forward in addressing household air pollution caused by polluting cookfuels.

The impetus for the MHFW report came from recent assessments on the burden of disease that highlight the scale of air pollution–related health impacts (Lim et al. 2012, Forouzanfar et al. 2015) and the need to address dual burdens from ambient and household air pollution in the nation (Balakrishnan et al. 2014). Around 1.5 million premature deaths in India—deaths due to a range of acute and chronic health conditions—are attributable annually to the indoor and outdoor exposures of the population to air pollution (IHME 2015). This places air pollution near or at the top of the list of all known risk factors for ill health in the country, above high blood pressure, smoking, child and maternal malnutrition, and risk factors for diabetes (IHME 2015). Although management of the latter risks has long been part of national health programs managed by the MHFW, the report makes a compelling case for a major national cross-ministerial effort directed at air pollution, to be spearheaded by the ministry. We describe here the salient recommendations of the report.

The health burden from air pollution comes from outdoor exposures in both urban and rural areas as well as from within households. In fact, approximately 170 million households, primarily in rural areas, are exposed indoors and near the household to pollution resulting from poor combustion of solid fuels such as wood, crop residues, and dung in traditional cookstoves (Bonjour et al. 2013). The result is more premature deaths from this cause (> 0.9 million annually) than in any other country (IHME 2015). Unfortunately, in spite of significant economic development and consequent growth in the use of clean fuels in some populations, the number of people using solid fuels in India does not seem to have changed significantly in the past 30 years (Bonjour et al. 2013), although it now constitutes a smaller fraction of the overall population. Vehicles and power plants—key contributors to urban ambient air pollution—historically have been the only sources to be addressed in national ambient air quality management efforts. The report addresses both household (indoor) and outdoor air pollution in an integrative fashion instead of treating them as separate issues—the first time by a government agency, to our knowledge. This is especially relevant given that household cookstoves also contribute an estimated 25% of ambient air pollution in India (Chafe et al. 2014).

Notably and importantly, the report invokes a new paradigm of exposure management instead of concentration management as a national air pollution control strategy, and prioritizes policies and actions accordingly to achieve substantially enhanced cost effectiveness and speed in achieving health benefits. From a health perspective, what matters is not just the absolute volume of emissions but how much of the pollution is breathed in by individuals, defined sometimes as the “intake fraction” (Bennett et al. 2002). With advances in understanding and capabilities that have come from new monitoring technologies, digital data management, remote sensing, and associated modeling, it is now possible to undertake such exposure apportionment in India. Several potential approaches can be used to rank sources that are most proximate to the population (such as stoves, vehicles, and neighborhood trash burning) as highest priority, since these produce the highest exposures per unit emissions. It implies somewhat different strategies for monitoring than have been used in other countries—for example, placing more ambient monitors in rural as well as urban areas to better follow population exposures (Balakrishnan et al. 2014).

The report provides a health-based argument for intensifying efforts to control household air pollution and to expand access to truly clean sources of household energy, shifting the focus away from just promoting so-called “improved” technologies that have not proven to be health protective (MNRE 2016). It also promotes a new research agenda to apply the range of modeling, networking, and sensing tools now becoming available to assess in specific places the impact of specific source types on exposure rather than just on concentrations (NRC 2012).

The report also highlights that reducing air pollution to achieve health goals will require actions across a range of government agencies, but that it can—and should—be led by health ministries (as has been the case for smoking and sanitation, for example). A clearer recognition of the problem and articulation of possible pollution control strategies can cause other ministries to start taking steps on their own, as has been the case with the Ministry of Petroleum and Natural Gas, which has taken a number of innovative steps to expand access to clean cooking gas to the poor (see, for example, Tripathi et al. 2015). Health ministries can also deploy their own assets to mitigate the impacts of air pollution through appropriate training, treatment, and information dissemination. In India, the MHFW has a million local health workers, tens of thousands of hospitals and clinics, and thousands of institutions that train health professionals, as well as extensive infrastructure through the Rural Health Mission and state governments. Health ministries and medical doctors also retain unique status in most countries as credible sources of information to influence the public, media, and policy makers.

All in all, the integrative exposure management–driven approach taken by the MHFW report will not only help address this major problem in an efficient and effective manner, but also serve as a model for other developing countries that likewise need to manage health impacts from a combination of household and outdoor air pollution.

The report is pioneering particularly by virtue of its coming from a ministry of health. Ever since the widespread recognition of the hazards of environmental pollution in the 1950s, actions on air pollution have been nearly universally managed by environment agencies. Although health is commonly considered as a criterion for setting regulatory standards, air pollution has been considered neither in the same landscape as all the other important risk factors that affect national health (most of which lie in completely different sectors), nor in the context of the special assets of the health sector that can be brought to bear on air pollution control. Even though air pollution is a major threat, of course, a resource-constrained country such as India has many other health challenges to address as well, and needs to carefully weigh the relative benefits of actions across sectors.

With 10 of the dirtiest 20 cities in the world (WHO 2016) and 700 million people caught in the “*chulha* trap,” i.e., still lacking access to clean fuels for cooking (Smith and Sagar 2014), India is in the throes of a silent health crisis due to air pollution that has become the greatest of any major country in the world both in total and per capita (twice that of China) (IHME 2015). The extreme nature of the situation requires major initiatives and innovative cross-sectoral approaches to organize the monitoring and evaluation of targeted controls. India’s MHFW has set the ball in motion by providing a pioneering framework for addressing air pollution impacts for both rural and urban populations.
